# Health consultations at a performing arts health centre among classical music students based on electronic health record data: a cross-sectional study

**DOI:** 10.3389/fpsyg.2024.1245505

**Published:** 2024-04-02

**Authors:** Suze Steemers, Rogier M. van Rijn, Marienke van Middelkoop, Sita M. A. Bierma-Zeinstra, Janine H. Stubbe

**Affiliations:** ^1^Codarts Rotterdam, University of the Arts, Rotterdam, Netherlands; ^2^Department of General Practice, Erasmus MC University Medical Center Rotterdam, Rotterdam, Netherlands; ^3^PErforming Artist and Athlete Research Lab (PEARL), Rotterdam, Netherlands

**Keywords:** electronic health record, health problems, classical music students, injury, stress, performance improvement

## Abstract

Physical and mental health problems in music students are usually identified using self-reported data. The use of an Electronic Health Record database can avoid biases to give an overview of the extent of health problems in this population. Therefore, the aim of this study is to gain insight into both physical and mental health related issues in classical music students using this type of data collection. An Electronic Health Record database including five years of data from a Performing Arts Health Centre (PAHC) of a university or the arts, was used to analyze the number and characteristics of health consultations in music students. The total number of students, health consultations and the average number of visits per health consultation were calculated over five years. Furthermore, numbers and percentages of both physical and mental health consultations were registered. Also, the number and percentage of health consultations per instrument group were compared to the proportion of each instrument group within the specific sample. Over a period of five years, 230 students visited the PAHC and 417 health consultations were reported. 43.5% of the students who visited a health professional indicated at least one physical health consultation, 29.1% at least one mental health consultation and 27.4% at least one in both categories. An injury was the most frequently registered physical health consultation (40.2%), followed by performance improvement (9.8%) and stress (9.6%). Voice students registered relatively most health consultations. As far as we know, this is the first cross-sectional study using Electronic Health Record data from a PAHC to gain insight into both physical and mental health related issues in a population of classical music students. Looking at the variety of health consultations registered in the database, a multidisciplinary team and multidisciplinary approach are necessary to meet the needs of the students in terms of physical and mental health support and performance improvement.

## Introduction

Music is a discipline that is physically and mentally demanding. The workload in higher music education and in the professional work field is high due to many hours of daily practice, repetitive movement and ergonomically challenging postures needed to be able to play the instrument (Paarup et al., 2011; Rickert et al., 2013; Kenny and Ackermann, 2015; Silva et al., 2015; Kok et al., 2016). Furthermore, it requires the control of complex interactions between musicality, expressivity and neuromotor control (Silva et al., 2015). These demands make musicians and music students prone to physical and mental complaints. Even in the beginning of their career, music students already experience all kinds of physical complaints including pain in the shoulders, back and neck (Paarup et al., 2011). Psychological issues like stress, anxiety, and poor mental health (Ioannou et al., 2018; Steemers et al., 2020b; Vaag et al., 2021) are common in this population as well. Moreover, anxiety and depression symptoms are more present in the student population of musicians compared to the general student population (Vaag et al., 2021). Insight into physical and mental health is therefore essential to develop suitable preventive methods for this population (van Mechelen et al., 1992).

Physical and mental health problems are usually identified using self-reported data, e.g., surveys (Paarup et al., 2011; Baadjou et al., 2015; Kenny and Ackermann, 2015; Vaag et al., 2021). While someone can describe their own complaints and experiences in much detail, the use of self-reported data can be accompanied by some risks. van Winden and colleagues used self-reported outcomes to investigate the extent and characteristics of injuries in contemporary dance students (van Winden et al., 2019). The authors concluded that self-reported data are subjective and therefore it is difficult to provide an overview of specific medical diagnoses in their study sample since most performing arts students lacked medical expertise. Accordingly, they recommended to involve health professionals with extensive knowledge of the working field to provide medical diagnoses.

Moreover, many studies using methods with self-reported surveys, including retrospective study designs, suffer from response bias. When using a retrospective study design, in which participants need to recall their complaints over a specific period of time, recall bias can occur. In a study focusing on the development and validation of a registration system for sports injuries, Clarsen and colleagues recommended to use a retrospective period of seven days or at least no more than four weeks (Clarsen et al., 2013). Furthermore, other types of response bias are prevalent in self-reported data: this can include for example social-desirability bias or selection bias, which is mostly the case in a low response rate, resulting in an overestimation of the studied phenomenon (Etter and Perneger, 1997; Rosenman et al., 2011). To establish the actual extent and severity of health problems (van Mechelen et al., 1992), beside self-reported data, other types of data collection are necessary as well.

Electronic Health Record (EHR) data do not suffer from these types of biases, because health professionals collect the health information of patients during their visits. In dance medicine research, EHR databases are already used to determine injury prevalence and incidence and to develop injury prevention strategies (Vassallo et al., 2017; Katakura et al., 2023). In sports, mainly in football, large EHR databases to gain insight into injury incidences are available as well (Mack et al., 2020; Anderson et al., 2021; Inclan et al., 2023). Studies using EHR data to provide an overview of health-related issues in classical music students are scarce (Manchester, 1988; Manchester and Cayea, 1998; Niarchou et al., 2021; Zalpour et al., 2021). Zalpour and colleagues investigated data from physiotherapy consultation hours for musicians in Germany (Zalpour et al., 2021). A broad population of musicians could visit these consultation hours, but the clinic was mostly visited by music students for whom these visits were free of charge. The authors concluded that the most injured body parts were the spine and upper extremities. Instrumentalists had a higher risk of getting injured compared to non-instrumentalists (singing and musical). They also recommended an interdisciplinary approach to support musicians on all aspects of health. In a study of Niarchou and colleagues, EHR data were used to compare physical and mental health problems of 9,803 musicians with a control group. They concluded that certain problems, e.g., disease of the vocal cord, hearing loss, anxiety disorders and joint pain, were more prevalent in the group of musicians than in the control group (Niarchou et al., 2021). Self-reporting methods are widely used in performing arts medicine studies and not many studies used EHR data to study the number and characteristics of physical and mental complaints (Niarchou et al., 2021; Zalpour et al., 2021).

To our knowledge, information regarding health in classical music students is usually provided by self-reported data and information diagnosed by a team of health professionals is lacking. The variety of biases that is associated with self-report studies can be avoided by using Electronic Health Record data. Therefore, the aim of our study is to gain insight into numbers and characteristics of both physical and mental health consultations using Electronic Health Record (EHR) data of classical music students. As far as we know, this is the first study using an EHR database from a Performing Arts Health Centre (PAHC), including both physical and mental health in classical music students.

## Materials and methods

### Design

Since August 2016, Codarts Rotterdam, University of the Arts, has been using an EHR to register health consultations. These health consultations were registered by health professionals from Codarts’ Performing Arts Health Centre (PAHC). Anonymized data from August 2016 until December 2021 were used in the current cross-sectional study. PAHC consists of three physiotherapists, two student psychologists (fully-trained psychologists treating students as their clients), a (sports) dietician, a performance coach, a hearing specialist and a voice/speech therapist. This health team is available for all Codarts students, i.e., dance, music and circus departments. The main goal of PAHC is to provide students with support in terms of injury prevention, performance improvement (in terms of health) and general wellbeing. The physiotherapists and student psychologists are available four days a week and the voice/speech therapist is available one day a week. The number of available hours of these health professionals was based on the needs of the students and this was established in collaboration with the different departments, management, teachers and students. The facilities of PAHC are part of the student’s education and therefore free of charge for all Codarts students. PAHC supports students in terms of prevention and short-term complaints. In case of long-term treatment or more extensive treatment, students usually come to the PAHC for a first visit but will then be referred to PAHC’s external medical network.

For the current paper we used a cross-sectional study design using a database with health consultations including students from the classical music department. The data were registered by PAHC’s physiotherapists, student psychologists and voice/speech therapist.

### Team Around The Artist application

The Team Around The Artist (TATA) application is an online registration tool aiming to monitor consultations of students who use the PAHC facilities. In TATA, health professionals register information about the health consultations and number of visits in a specifically developed format. This EHR data system was developed in extensive collaboration with its users and is adapted to the needs of the different health professionals using TATA. The use of TATA is discussed on a regular basis, at least 4 times a year, with the health professionals, researchers and project leaders involved. The TATA formats used by the various health professionals, which can be found in Appendices 1–3, are under constant development to improve and optimize the system. The list of request types of health consultations was determined based on the experience of the health professionals in the previous years.

### Population

The Bachelor of Music at Codarts, University of the Arts, Rotterdam, is a four-year studies in which students mainly focus on one instrument. After graduating, students can audition for the Master of Music studies, a two-year studies in which artistic research plays a major role. The classical music department offers a wide variety of main instruments, including for example violin, voice and composition. In the current study, classical music students registered with at least one health consultation in TATA were included. Since the classical music department has a different type of curriculum than other music studies at Codarts (e.g., pop or jazz music) and may therefore present different types of health related issues, we chose to include specifically classical music students.

This study was approved by the Medical Ethics Committee (MEC-2019-0163) of the Erasmus MC University Medical Center Rotterdam, Netherlands. Furthermore, all data were anonymized before the analysis.

### Statistical analysis

Statistical analyses were conducted using SPSS (version 28). In case of missing details concerning the student’s main instrument, visited health professional or type of health consultation, health consultations were excluded from the analysis. The percentage PAHC users in the academic year 2020–2021 was calculated as a percentage of the total number of classical music students, using the most reliable Codarts student database (2020–2021) from the administration department.

Baseline characteristics of the population of classical music students registered in TATA were calculated with descriptive statistics. Means and standard deviations (SD) or numbers and percentages (%) were included. In addition, descriptive statistics were used to give an overview of the total number of health consultations, health consultations per student and number of visits per health consultation. For the analysis, all health consultations were divided in two categories: ‘physical health consultations’ and ‘mental health consultations’. Numbers and percentages of both physical and mental health consultations were registered. In case of physical health consultations, if more than one body region was registered by the health professional, the body region that was registered first was included in the analysis. Also, the number and percentage of health consultations per instrument group were calculated and compared to the representation of each instrument group within the classical music department at Codarts.

## Results

### Study population

In Table 1, more information is presented about the number of enrolled classical music students, students who did or did not visit the PAHC, their gender and whether they followed a Bachelor or Master program. As stated in the methods section, the number of enrolled classical music students from the study year 2020–2021 was used for calculations as this was the most reliable database available. The total number of enrolled students was 168 of which 50 students used the PAHC facilities. Most of these students were female (*n* = 35) and Master students (*n* = 27).

**Table 1:** Baseline characteristics of Codarts students from study year 2020–2021.

	Enrolled classical music students 2020–2021	Students using PAHC facilities 2020–2021	Students not using PAHC facilities 2020–2021
Total	168	50	118
Sex			
Women	83	35	48
Men	85	15	70
Bachelor/Master			
Bachelor	114	23	91
Master	54	27	27

In Table 2, all baseline characteristics of classical music students registered in TATA are presented. In total, 230 Bachelor and Master classical music students were registered in TATA and included in the analysis. The majority of the included students were women. Most of the students followed the Bachelor program. Mean age of all included students was 23 years.

**Table 2:** Baseline characteristics of Codarts students using the PAHC facilities shown as means (±SD), percentages or numbers.

	Mean or number
Age (yrs)	23.06 ± 3.28
Average % of classical music students using PAHC facilities	29.8%
Sex (%)	
Women	58.7% (*n* = 135)
Men	41.3% (*n* = 95)
Bachelor/Master	
Bachelor	63.9% (*n* = 147)
Master	36.1% (*n* = 83)
Instrument groups	
String instruments	41.3% (*n* = 95)
Voice	15.2% (*n* = 35)
Woodwind instruments	12.2% (*n* = 28)
Piano & organ	9.6% (*n* = 22)
Composition/direction	8.3% (*n* = 19)
Brass instruments	7.0% (*n* = 16)
Percussion	4.8% (*n* = 11)
Other	1.7% (*n* = 4)

### Health consultations

On the total of 230 students, 417 health consultations were reported and included in the analysis.

Nine health consultations were excluded from the analysis due to missing details (e.g., unknown instrument type or missing type of health consultation). The average number of health consultations per student using PAHC facilities was 1.81, with a minimum of 1 and a maximum of 8 health consultations. The average number of visits per health consultations was 3.27 (SD ± 3.05, range 1–22 visits).

29.8% of the total classical music population at Codarts used the PAHC facilities. Of all 230 students, 100 students reported at least one physical health consultation with one of the PAHC professionals. Of the total of 230 students, 67 students reported at least one mental health consultation. 63 out of 230 students reported at least one physical and at least one mental health consultation. This results in, respectively, 43.5, 29.1 and 27.4% of all reported health consultations.

### Physical health consultations

A more detailed overview of all types of physical health consultations is shown in Table 3. In total, 251 physical health consultations were identified in TATA. This was 60.2% of all registered health consultations. The most prominently registered health consultation in the EHR database overall was injury (40.3% of all health consultations), followed by performance improvement (9.8%).

**Table 3:** Number of registrations per type of physical health consultation.

Types of physical health consultations	Number of registrations	% of total number of health consultations
Injury	168	40.3%
Performance improvement	41	9.8%
Prevention	10	2.4%
Other	9	2.2%
Vocal cords guidance	7	1.7%
Voice overload	6	1.4%
Jaw guidance	5	1.2%
Voice fatigue/ relaxation	4	1.0%
Breathing	1	0.2%
Total	251	60.2%

Table 4 presents the injured body parts. The most injured body part was the neck/shoulder region (41.7%), followed by the arm region (26.8%). In nine health consultations, the specific body region was not registered correctly. Looking into the nature of these injuries (Table 5), overload was most frequently registered by the physiotherapists as onset of injuries (*n* = 100).

**Table 4:** Number of registrations per type of injury.

Injured body region	Number of injuries	% of total number of injuries
Neck/shoulders	70	41.7%
Arm region (arm, hand/wrist/elbow/fingers)	45	26.8%
Back/vertebrae	18	10.7%
Lower extremities	14	8.3%
Other (e.g., general pain complaints, hip, jaw)	12	7.1%
Missing	9	5.4%
Total number of injuries	168	100%

**Table 5:** Nature of injuries, registered by physiotherapists.

Nature of injury	Number of injuries
Overuse	100
Trauma	20
External factor/other	10
Unknown	38
Total	168

### Mental health consultations

In TATA, 166 mental health consultations were registered by the health professionals (Table 6). This was 39.8% of all indicated health consultations. Mental health support was mostly provided for stress (9.6% of all health consultations), followed by anxiety (4.6%), mood consultations (4.1%) and self-image (3.8%).

**Table 6:** Number of registrations per type of mental health consultation.

Type of mental health consultation	Number of registrations	% of total number of health consultations
Stress	40	9.6%
Anxiety	19	4.6%
Mood	17	4.1%
Self-image	16	3.8%
Family/relation	15	3.6%
Study behaviour/skills	15	3.6%
Perfectionism	14	3.4%
Motivation	9	2.2%
Overworked/overloaded	8	1.9%
Eating problems	5	1.2%
Dealing with injury	3	0.7%
Mourning	3	0.7%
Emotion regulation	1	0.2%
Development issues (e.g., ADHD, DCD)	1	0.2%
Total	166	39.8%

### Instrument groups

Of all included instrument groups, string instruments were most frequently represented in the EHR database with 37.9%, followed by singers (22.3%) and woodwind instruments (11.3%). However, the group of string instruments is also the largest instrument group of students within the classical music department of Codarts (39.1%). In Table 7 the percentage of health consultations and the percentage of the instrument groups as part of the total classical music department are shown. When investigating the number of health consultations in comparison to the number of students within each instrument group, the voice students registered relatively most health consultations (22.3%). Looking more specifically into the type of health consultation that was most prominent in each group, it becomes clear that injury was most frequently mentioned. Exception is the group of singers in which performance improvement was indicated in 24 out of 93 health consultations. The composition/direction group suffered the least from health consultations, compared to the number of students within their instrument group.

**Table 7:** Physical and mental health consultations per instrument group.

Health consultation → Instrument group ↓	Physical health consultation (*n*)	Mental health consultation (*n*)	Total number of health consultations per instrument group (*n*)	Most frequently registered health consultation	% of total number of health consultations	% of total classical music students
String instruments	92	66	158	Injury	37.9%	39.1%
Voice	31	62	93	Performance improvement	22.3%	10.7%
Woodwind instruments	25	22	47	Injury	11.3%	11.2%
Piano and organ	17	24	41	Injury	9.8%	11.6%
Percussion	13	8	21	Injury	5%	4.7%
Composition/direction	12	15	27	Injury	6.5%	11.6%
Brass instruments	10	12	22	Injury	5.3%	9.9%
Other	3	5	8	Injury	1.9%	1.7%

## Discussion

The aim of this study was to investigate the number and characteristics of health consultations using an EHR database of a Performing Arts Health Centre among a population of classical music students. The data were collected over a period of 5 years.

The main findings of the current study were that 43.5% of the students who visited a PAHC professional indicated at least one physical health consultation, 29.1% at least one mental health consultation and 27.4% at least one in both categories. An injury was the most frequently registered physical health consultation, representing 40.2% of all included health consultations. These injuries were mostly overuse injuries. Injury was followed by performance improvement (9.8%). Stress was mostly mentioned as a mental health consultation with 9.6% of all registered health consultations.

In agreement with other studies, injuries are a common health problem in musicians (Zaza et al., 1998; Silva et al., 2015; Kok et al., 2016; Viljamaa et al., 2017; Ballenberger et al., 2018). Kok and colleagues, showed in an extensive review that lifetime prevalences range between 62–93% (Kok et al., 2016). Most of the physical health problems mentioned by musicians are located in the upper extremities (Sousa et al., 1998; Kok et al., 2016; Viljamaa et al., 2017) and injuries are usually associated with overuse (Viljamaa et al., 2017; Yang et al., 2021). However, as already stated, most methods use self-reported surveys risking selection bias which may cause an overestimation of the actual number of health related issues (Etter and Perneger, 1997; Rosenman et al., 2011). EHR database including data from health professionals can provide more insight into the actual medical diagnoses. Furthermore, a team of health professionals can use this information for suitable support and guidance in recovery or optimizing a healthy way of playing music. The influence of injury definitions on outcomes in classical music students was already investigated by our group (Steemers et al., 2020a). We found a large discrepancy between the percentage of using an all musculoskeletal complaint definition (96.6%) compared to a medical attention (17.2%) and time-loss definition (13.8%), all used in the same population. It was concluded that the choice of definition needs to be adapted to the practical implications of the outcomes, for example, orchestra management might be most interested in time-loss injuries for the purpose of replacing musicians for a concert. Therefore, collecting various forms of data, both self-reported and medically diagnosed, provide the most complete overview and possibilities for different practical implications.

Our results also showed that most students using the PAHC facilities were female (58.7%). This is in line with other studies indicating that female musicians appear to be more likely to develop injuries compared to male musicians (Manchester and Cayea, 1998; Baadjou et al., 2016; Kok et al., 2016; Borger et al., 2021). However, another explanation for the higher percentage of female music students visiting PAHC could also be that they tend to ask for support in an earlier stage. It would be interesting to investigate this in future research and provide more details about the intake consultations of female and male when visiting a health professional.

In agreement with other studies about high workload and occupational demands of musicians, the current study has shown that stress is an important topic registered by the student psychologists in TATA (Rickert et al., 2013; Willis et al., 2019). Araujo and colleagues concluded in their research that music students do not often engage in stress management behaviors (Araújo et al., 2017). They emphasize that psychological health education early in the musicians career to prevent mental complaints is imperative. Although attention to the topic of mental health is growing among musicians and in higher music education, only a few studies have focused on the size and the severity of this specific issue for musicians or music students (Ballenberger et al., 2018; van Fenema et al., 2018; Jääskeläinen et al., 2020). Beside stress, performance improvement was also an important topic reported in the TATA database. Since the performing experiences of music students are less then professionals, it may be useful to invest in specific practice environments for students to enhance their performance. However, settings like concert halls are usually expensive. Aufegger and colleagues looked into training and learning possibilities for music students, using simulation training environments: a recital with a virtual audience and the simulation of an audition setting with virtual judges were developed in which students could perform (Aufegger et al., 2017). The experiences were analyzed qualitatively using focus group interviews and written input. The participants indicated that virtual performance environments can be useful and effective tools for practicing and enhancing performance and simultaneously working on mental skills and performance preparation. Williamon and colleagues used a similar virtual addition setting for music students and measured both state anxiety and heart rate variability (Williamon et al., 2014). The results were compared with a real audition setting and no differences were found between the real and virtual setting on these measures, suggesting that the virtual setting could create a similar stress response when compared to the real setting. Implementing such virtual practice settings for music students could consequently be an interesting option for higher music education aiming for performance enhancement and stress reduction.

Codarts’ PAHC not only offers support for health related issues, but also questions about prevention and performance improvement related to health were addressed by the students. The fact that music students in the current population sought help in terms of both physical and mental health problems as well as questions about performance improvement, underpins the importance of a multidisciplinary team with a focus on prevention and optimal performance instead of a mainly curative approach. This is in line with Araujo and colleagues stating that a support team including a variety of music education specialists “commit collectively to the development of mechanisms that support students to build psychological resilience to achieve optimal health and wellbeing and optimize their practice and performance” (Araújo et al., 2017). To meet the needs of music students in terms of healthy performance, future research could investigate the most effective composition of health professionals in such a support team.

Taking the proportion of various instrument groups within the classical music department into account, voice students used relatively most health support from the PAHC professionals. Literature about physical and/or mental health in musicians mainly focuses on instrumentalists and studies looking into singers or voice students focusing on this broad topic are scarce (Jahn, 2009). Available studies usually investigate voice-specific health issues or treatment rather than more general physical and mental health aspects within this population (Haben, 2012; Caffier et al., 2017). The current study reveals that this type of student also experiences health problems like injuries or mental health issues. It appears to be that this specific group of students also focuses on performance improvement instead of mere treatment for health complaints. Within Codarts, the voice teachers department is closely involved in the PAHC facilities. Teachers focus on healthy performance within specific ‘optimal performance classes’ in the voice department. Therefore, an explanation of this higher percentage of health support may be that these students are more aware of the importance of healthy performance than other students.

### Strengths and limitations

As far as we know, this is the first study using Electronic Health Record data from a Performing Arts Health Centre to gain insight into health support in a population of classical music students. Compared to the mostly self-reported data used in other studies, this study provides insight in both physical and mental health consultations diagnosed by health professionals and therefore a more accurate insight into issues within this population. The variety of indicated health consultations emphasizes the need for the availability of a multidisciplinary team during their studies to meet the needs of music students.

One of the limitations of this study is that we do not have sufficient insight into the external medical support that students might seek. Some students already have a physiotherapist when they start studying at Codarts and might prefer to seek for support there. Also, our PAHC focuses mainly on prevention and short-term injuries. When injuries are more severe or long-term treatment is necessary, students are referred to the external network of (para)medical experts. While PAHC professionals usually keep in touch with these experts to track the progression of the students, and most students usually visit the PAHC before they are referred to the external network, this may cause a slight underestimation of the number of visits necessary for treatment. However, all PAHC facilities at Codarts are free of charge and the external network is not. Therefore we assume that most Codarts students will visit PAHC professionals first before they seek help within the external medical network.

Secondly, other studies suggest that musicians tend to conceal their injuries or other health problems and this seems to be the norm (Rickert et al., 2014; Roos et al., 2021). Fear to lose one’s job, identity issues and lack of perceived support play an important role. According to Matei and Philips, there still appears to be a stigma around pain and anxiety and musicians usually assume that pain is caused by instrumental technique (Matei and Phillips, 2023). Pain beliefs still appear to be part of the cultural norm in musicians. Physical and mental support registered in the EHR database might therefore be an underestimation of the actual number of physical and mental health issues and higher music education needs to be aware of this when developing preventive programs for their students.

Thirdly, the TATA system is still under development. This means that the registration formats which are presented in Appendices 1–3, were not fully available during the first years of data collection. Therefore, we miss some details, e.g., on reference to external medical network or performance status, which would elaborate more on the health consultations and their consequences for the students. Furthermore, not all fields in our TATA system are standardized, using a set collection of options that could be selected. This is for example the case in the description of the nature of injuries. Also, although extensive discussion with the health professionals led to clear request types, clear definitions of the request types are lacking and should be developed for future research and optimal use of our TATA system. A study with a focus on definitions and details of health consultations and a standardization of the selected options in such an EHR would be interesting for future research.

Lastly, during some of the period in which these data were collected, corona measures to reduce the spread of the infection were in place. Students were not able to go to school for a few months (March–May 2020). The PAHC visits were therefore harder to organize and most of them took place online or did not take place at all. This might have caused an underestimation of the number of health consultations in that specific period. According to a study by Stubbe et al. (2021), performing arts students, including dance students, circus arts students and musical theater students, experienced decreased stress levels, increased sleep quality and increased mental health complaints during this specific period with corona measures in the Netherlands (Stubbe et al., 2021). Although the results were based on self-report and classical music students were not included in the database, it might be interesting to look into more details what this specific period meant for this population including both self-report and medical data.

## Conclusion

As far as we know, this is the first study using Electronic Health Record data of a Performing Arts Health Centre to gain insight into the number and characteristics of physical and mental health consultations in a population of classical music students. Injuries, stress and performance improvement were most frequently registered as topics for which students sought health support. An EHR database can provide more insight into medical diagnoses provided by health professionals. This information is essential for optimal guidance and support of musicians in healthy practicing and performing. A multidisciplinary team and multidisciplinary approach including for example virtual performance settings are necessary to meet the needs of the students in terms of physical and mental health support and performance enhancement.

## Data availability statement

The data analyzed in this study is subject to the following licenses/restrictions: the datasets presented in this article are not readily available because the dataset contains medical data. Requests to access these datasets should be directed to ssteemers@codarts.nl or jhstubbe@codarts.nl.

## Ethics statement

The studies involving humans were approved by Medical Ethics Committee (MEC-2019-0163) of the Erasmus MC University Medical Center Rotterdam, Netherlands. The studies were conducted in accordance with the local legislation and institutional requirements. Written informed consent for participation was not required from the participants or the participants’ legal guardians/next of kin because data was anonymized before analysis.

## Author contributions

SS initiated the study, analyzed the data, and wrote the first draft of the manuscript. JS and RR participated in drafting the article and contributed to the data analysis. RR, JS, MM, and SB-Z reviewed and edited the article. All authors contributed to the article and approved the submitted version.

## Supplementary material

Appendix 1 Flowchart for creating a new indication in the online registration tool TATA for physiotherapist.

Appendix 2 Flowchart for creating a new indication in the online registration tool TATA for psychologist.

Appendix 3 Flowchart for creating a new indication in the online registration tool TATA for voice therapist.
