# Supplementary figures and images for: Health consultations at a performing arts health centre among classical music students based on electronic health record data: a cross-sectional study
Source: Front Psychol. 2024 Apr 2;15:1245505. doi: 10.3389/fpsyg.2024.1245505 (PMC11020097; doi:10.3389/fpsyg.2024.1245505)

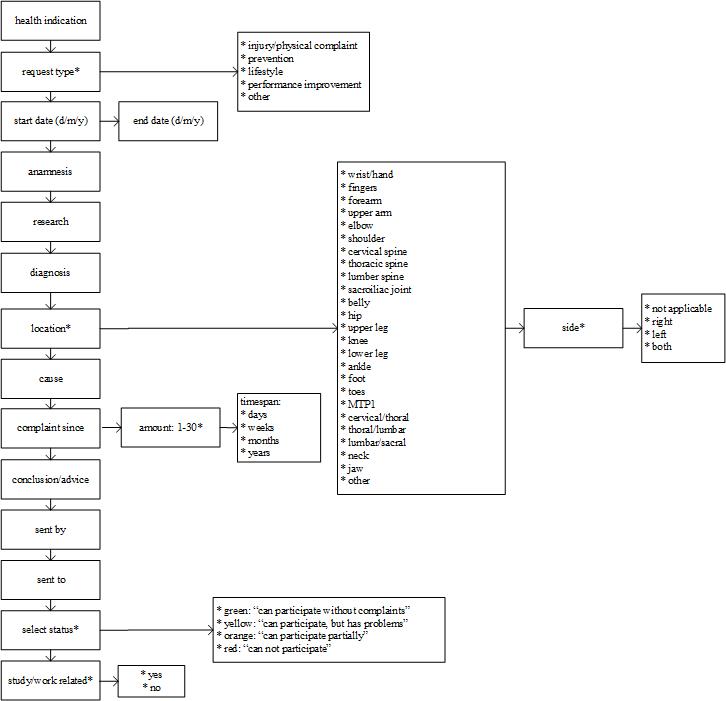

Supplement: Appendix 1 — Flowchart for creating a new indication in the online registration tool TATA for physiotherapist. [file Image_1.jpg]

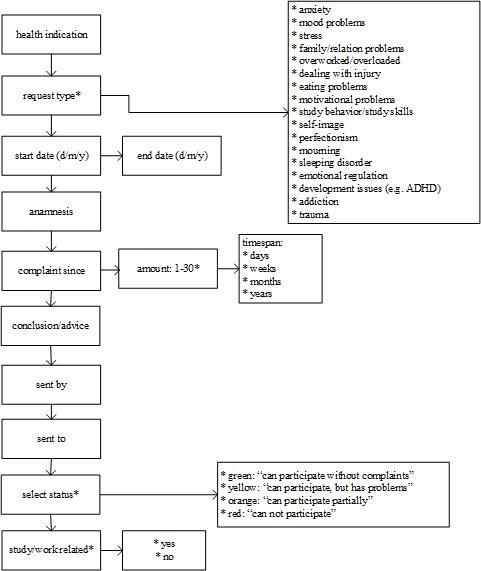

Supplement: Appendix 2 — Flowchart for creating a new indication in the online registration tool TATA for psychologist. [file Image_2.jpg]

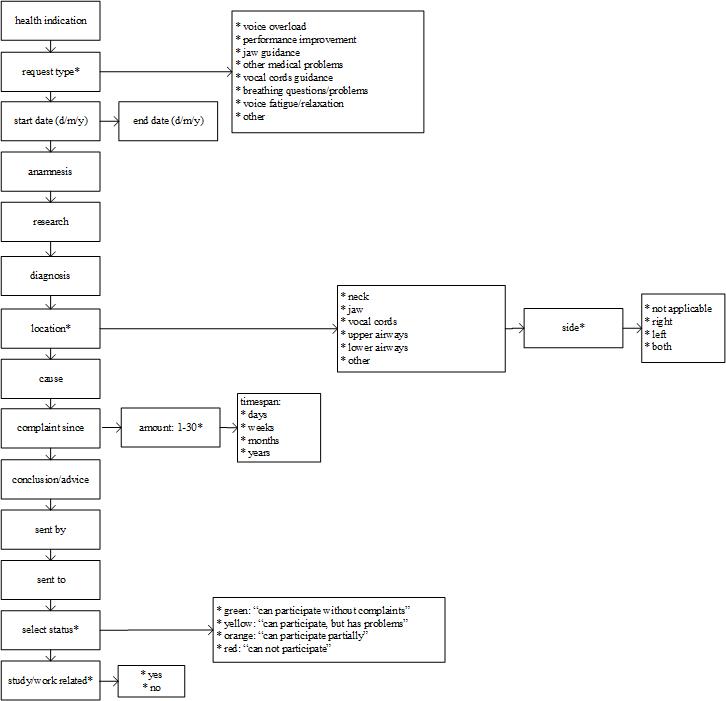

Supplement: Appendix 3 — Flowchart for creating a new indication in the online registration tool TATA for voice therapist. [file Image_3.jpg]
